# Histone variant H2A.Z promotes meiotic chromosome axis organization in *Saccharomyces cerevisiae*

**DOI:** 10.1093/g3journal/jkac128

**Published:** 2022-05-24

**Authors:** Lorencia Chigweshe, Amy J MacQueen, Scott G Holmes

**Affiliations:** Department of Molecular Biology and Biochemistry, Wesleyan University, Middletown, CT 06459, USA; Department of Molecular Biology and Biochemistry, Wesleyan University, Middletown, CT 06459, USA; Department of Molecular Biology and Biochemistry, Wesleyan University, Middletown, CT 06459, USA

**Keywords:** meiosis, chromatin, histone H1, H2A.Z

## Abstract

Progression through meiosis is associated with significant reorganization of chromosome structure, regulated in part by changes in histones and chromatin. Prior studies observed defects in meiotic progression in yeast strains lacking the linker histone H1 or variant histone H2A.Z. To further define the contributions of these chromatin factors, we have conducted genetic and cytological analysis of cells undergoing meiosis in the absence of H1 and H2A.Z. We find that a spore viability defect observed in strains lacking H2A.Z can be partially suppressed if cells also lack histone H1, while the combined loss of both H1 and H2A.Z is associated with elevated gene conversion events. Cytological analysis of Red1 and Rec8 staining patterns indicates that a subset of cells lacking H2A.Z fail to assemble a proper chromosome axis, and the staining pattern of the synaptonemal complex protein Zip1 in *htz1Δ/htz1Δ* cells mimics that of cells deficient for Rec8-dependent meiotic cohesion. Our results suggest a role for H2A.Z in the establishment or maintenance of the meiotic chromosome axis, possibly by promoting the efficient chromosome cohesion.

## Introduction

Meiosis is a specialized cellular differentiation program allowing diploid cells to follow a single cycle of DNA replication with 2 successive cell divisions, resulting in the generation of 4 haploid cells. In yeast and other organisms, meiosis is associated with changes in the structure of chromatin that influence the execution of a unique gene expression program, the creation and repair of DNA double-strand breaks, and the regulation of chromosome pairing and segregation patterns specific to meiosis (Govin and Berger 2009; Neiman 2011; Hunter 2015; Jaiswal *et al.* 2017; Hong *et al.* 2019). In yeast cells, sporulation is not associated with widespread changes in genome-wide nucleosome occupancy or positioning (Zhang *et al.* 2011), but progression through meiosis is correlated with specific alterations in the posttranslational modification of histones (Krishnamoorthy *et al.* 2006; Govin, Dorsey, *et al.* 2010; Govin, Schug, *et al.* 2010; Hu *et al.* 2015; Kniewel *et al.* 2017; reviewed in Govin and Berger 2009; Jaiswal *et al.* 2017; Wang *et al.* 2017). Meiosis is also influenced by the presence of linker histone H1 and the variant histone H2A.Z. Histone H1 is encoded by the *HHO1* gene in budding yeast, and its absence in vegetative cells is associated with relatively subtle phenotypes (Patterton *et al.* 1998; Downs *et al.* 2003; Veron *et al.* 2006; Li *et al.* 2008; Kowalski and Pałyga 2012; Lawrence *et al.* 2017). In yeast cells, diploids lacking H1 are reported to have mild defects in sporulation efficiency (Bryant *et al.* 2012) with spore viability and recombination efficiencies similar to those of wild-type cells (Bryant *et al.* 2012; Brush 2015). H1 may partner with the Ume6 repressor to limit the expression of early meiotic genes during vegetative growth, while enrichment of H1 during spore maturation may contribute to chromatin compaction (Bryant *et al.* 2012).

H2A.Z, encoded by the *HTZ1* gene in budding yeast, is a highly conserved variant of the canonical histone H2A; yeast cells lacking H2A.Z have a variety of defects in gene expression, DNA damage repair, and chromosome segregation (Santisteban *et al.* 2000; Meneghini *et al.* 2003; Krogan *et al.* 2004; Kalocsay *et al.* 2009; reviewed in Billon and Cote 2013; Giaimo *et al.* 2019). Fission yeast lacking H2A.Z are deficient in the formation of the double-strand DNA breaks that initiate meiotic recombination (Yamada *et al.* 2018), yet increases the frequency of crossovers at some loci (Yamada *et al.* 2020). No such defect in DNA break formation was observed in budding yeast cells lacking H2A.Z (Gonzalez-Arranz *et al.* 2018); however, *htz1Δ/htz1*Δ diploids were observed to show reduced sporulation efficiency and spore viability, had defects in resumption of progression through meiosis following activation of the meiotic recombination checkpoint, and exhibited reduced chromosome movement in meiotic prophase (Gonzalez-Arranz *et al.* 2018, 2020).

Successful meiosis depends on formation of a specialized chromosome axis that includes the Red1 and Hop1 proteins, and the meiosis-specific cohesin Rec8 (Hollingsworth *et al.* 1990; Smith and Roeder 1997; Klein *et al.* 1999; Zickler and Kleckner 1999). This axis is required for the subsequent assembly of the synaptonemal complex (SC), which facilitates the intimate association of homologous chromosomes and regulates the interhomolog recombination events that are essential for proper chromosome segregation at meiosis I (Page and Hawley 2003). The varied meiotic defects observed in yeast cells lacking either H1 or H2A.Z could stem from defects in the formation of chromosomal axes or the downstream process of SC assembly. In this study, we examined the individual and joint contributions of H1 and H2A.Z to meiotic prophase chromosomal events. Our study extends prior work indicating that loss of H1 or H2A.Z does not alter patterns of recombination during meiosis, demonstrates that loss of H1 can suppress the decrease in spore viability caused by the absence of H2A.Z, and identifies a chromosome axis defect in cells lacking H2A.Z. Our results suggest that in a subset of meiotic cells, H2A.Z plays a key role in establishing or stabilizing chromosome axes.

## Materials and methods

### Strains

All *Saccharomyces* *cerevisiae* strains used in this study are isogenic to BR1919-8B (Rockmill and Roeder 1998) and are described in Table 1. Strain YSH1496 was created by crossing haploid strains YSH1305 (*MATα*) and YSH1306 (*MATa*). Gene deletions in each haploid were performed via standard PCR-based gene disruptions (Wach *et al.* 1994) using MX plasmids as templates (Goldstein and McCusker 1999; Knop *et al.* 1999); haploids were then mated to create homozygous diploids. Diploids deleted for both *HTZ1* and *HHO1* were created by standard crosses and tetrad dissections. Strains YSH1496 (wild type), YSH1497 (*htz1Δ/htz1Δ*), YSH1498 (*hho1Δ/hho1Δ*), and YSH1515 (*htz1Δ/htz1Δ hho1Δ/hho1Δ*) were used for spore viability, sporulation efficiency, and recombination assays. These strains are heterozygous for markers on chromosomes III and VIII to facilitate recombination analysis, as illustrated in Fig. 2a (Voelkel-Meiman *et al.* 2016).

**Table 1.** Strains.

Strain	Genotype
BR1919	*MATa ura3-1 thr1-4 trp1-289 ade2-1 his4-260,519 leu2-3,112 LYS2*
YSH1305 (YAM3654)	*MATα ura3-1 ade2-1 trp1-289 leu2-3,112 lys2ΔNhe hphMX4@CEN3 ADE2@RAD18 natMX4@HMR TRP1@SPO11 spo13Δ::URA3 HIS4 THR1*
YSH1306 (YAM3738)	*MATa ura3-1 thr1-4 trp1-289 ade2-1 his4-260,519 leu2-3,112 lys2ΔNhe LYS2@cVIII (210Kb)*
YSH1496	*MATa lys2ΔNhe leu2-3,11 ade2-1 ura3-1 trp1-289 thr1-4 LYS2@cVIII* *MATα lys2ΔNhe leu2-3,11 ade2-1 ura3-1 trp1-289 THR1 cVIII* *his4-260,519 spo13Δ::URA3 hphMX4@CEN3 TRP1@SPO11 natMX4@HMR* *HIS4 SPO13 CEN3 SPO11 HMR*
YSH1497	YSH1496; *htz1Δ::kanMX4*/*htz1Δ::kanMX4*
YSH1498	YSH1496; *hho1Δ::kanMX4*/*hho1Δ::kanMX4*
YSH1515	YSH1496; *htz1Δ::kanMX4/htz1Δ::kanMX4 hho1Δ::kanMX4/hho1Δ::kanMX4*
YSH1304 (YAM805a)	*MATa ura3-1 thr1-4 trp1-289 ade2-1 his4-260,519 leu2-3,112 lys2ΔNhe ndt80Δ::LEU2*
YSH1307 (YAM2593)	*MATα ura3-1 thr1-4 trp1-289 ade2-1 his4-260,519 leu2-3,112 lys2ΔNhe zip1Δ::hphMX4 ECM11-13MYC-kanMX4 ndt80Δ::LEU2*
YSH1524	*MATa trp1-289 leu2-3,112 ade2-1 thr1-4 ndt80Δ::LEU2 lys2ΔNhe* *MATα trp1-289 leu2-3,112 ade2-1 thr1-4 ndt80Δ::LEU2 lys2ΔNhe* *his4-260,519 ECM11 HTZ1 HHO1* *his4-260,519 ECM11 HTZ1 HHO1*
YSH1525	YSH1504; *htz1Δ::natMX4/htz1Δ::natMX4*
YSH1526	YSH1504; *hho1Δ::natMX4/hho1Δ::natMX4*
YSH1527	YSH1504; *htz1Δ::natMX4/htz1Δ::natMX4 hho1Δ::natMX4/hho1Δ::natMX4*
YSH1616	*MATα trp1-289 leu2-3,112 ade2-1 thr1-4 ndt80Δ::LEU2 lys2* *MATa trp1-289 leu2-3,112 ade2-1 thr1-4 ndt80Δ::LEU2 lys2* *his4-260,519 ura3-1 REC8-MYC-kanMX4 HTZ1 HHO1* *his4-260,519 ura3-1 REC8 HTZ1 HHO1*
YSH1618	*MATα trp1-289 leu2-3,112 ade2-1 thr1-4 ndt80Δ::LEU2 lys2* *MATa trp1-289 leu2-3,112 ade2-1 thr1-4 ndt80Δ::LEU2 lys2* *his4-260,519 ura3-1 REC8 HTZ1 hho1Δ::natMX4* *his4-260,519 ura3-1 REC8-MYC-kanMX4 HTZ1 hho1Δ::natMX4*
YSH1620	*MATa trp1-289 leu2-3,112 ade2-1 thr1-4 ndt80Δ::LEU2 lys2* *MATα trp1-289 leu2-3,112 ade2-1 thr1-4 ndt80Δ::LEU2 lys2* *his4-260,519 ura3-1 REC8-MYC-kanMX4 htz1Δ::natMX4 HHO1* *his4-260,519 ura3-1 REC8 htz1Δ::natMX4 HHO1*

Diploids were induced to undergo meiosis on solid sporulation media (0.1% yeast extract and 1% potassium acetate) at 30°C for 5 days; cells were then assessed for sporulation efficiency, spore viability, and recombination efficiency as described (Voelkel-Meiman *et al.* 2016). Stahl Lab Online Tools (https://elizabethhousworth.com/StahlLabOnlineTools/) were used to calculate recombination efficiencies and crossover interference (Stahl and Lande 1995).

### Cytological analysis and imaging

Strains used for cytological analysis are homozygous *ndt80Δ::LEU2*; these diploids arrest at pachytene of prophase I. Diploid strains homozygous for specific gene deletions were created by first deleting the *HTZ1* and *HHO1* genes in haploid strains YSH1304 (*MATa*) and YSH1307 (*MATα*) followed by crosses. Strains YSH1524 (wild-type), YSH1525 (*htz1Δ/htz1Δ*), YSH1526 (*hho1Δ/hho1Δ*), and YSH1527 (*htz1Δ/htz1Δ hho1Δ/hho1Δ*) were used for cytological analysis. Diploids were induced to sporulate in liquid media at 30°C, and then, meiotic chromosomes were surface spread on glass slides as described (Rockmill 2009; Voelkel-Meiman *et al.* 2012). Spread chromosomes were immunostained as described (Voelkel-Meiman *et al.* 2019). Prior to imaging, 12 µL of glycerol-based mounting medium containing 1 mg/mL DAPI was added to each slide; a cover slip was then placed on top of each slide. Imaging was performed using the Deltavision RT imaging system (General Electric) adapted to an Olympus (IX71) microscope. Antibodies targeting SC transverse filament protein Zip1 and targeting the SC central element protein(s) Ecm11-Gmc2 (Voelkel-Meiman *et al.* 2019) were used to assess the SC structure. Random nuclei for each genotype were analyzed for their Zip1 and Ecm11-Gmc2 staining pattern and placed into one of 4 categories: linear (fully continuous lines of Zip1); dotty linear (mixture of Zip1 foci and short linear stretches); and dotty (foci only) (Voelkel-Meiman *et al.* 2012). Nuclei with a fuzzy/misty Zip1 pattern that is coincident with DAPI staining were placed into the “diffuse” category.

## Results

### Cells lacking H2A.Z have decreased spore viability

**Fig. 1.**
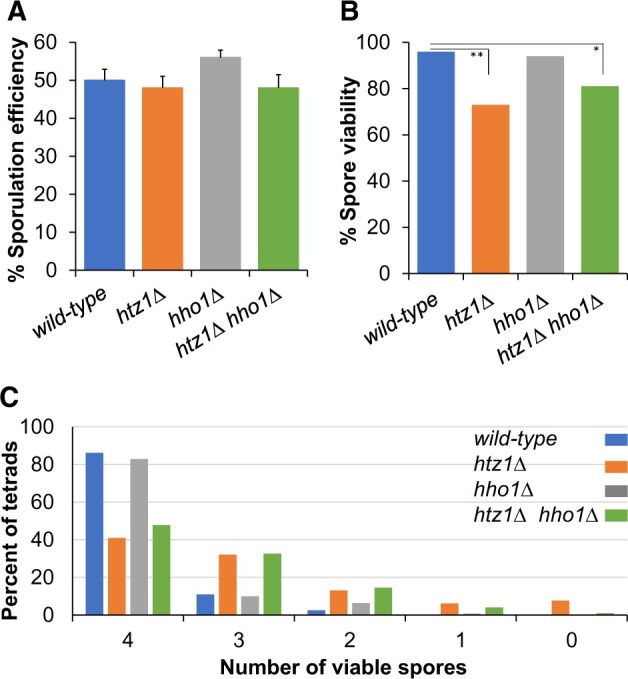
Sporulation efficiency and spore viability in the absence of H2A.Z or H1. a) Sporulation efficiency data was accumulated from 10 biological replicates for each of the indicated strains. A 2-tailed Student’s *t*-test was performed to analyze the differences between strains; error bars represent one standard error. No pairwise comparison of sporulation efficiency between different genotypes is significant. Numbers of cells analyzed and the *P*-value compared to wild type: wild type (*n* = 6,800); *htz1Δ* (*n* = 6,841, *P* = 0.50); *hho1Δ* (*n* = 6,795, *P* = 0.35); and *htz1Δhho1Δ* (*n* = 2,601, *P* = 0.24). b) Spore viability. The number of tetrads analyzed for each strain and *P*-values comparing values to wild type are as follows: wild type (*n* = 584); *htz1Δ* (*n* = 781, *P* = 5.96E−15); *hho1Δ* (*n* = 643, *P* = 0.0323); and *htz1Δ hho1Δ* (*n* = 869, *P* = 1.27E−13). The difference between the *htz1Δ* and *htz1Δ hho1Δ* strains is significant (*P* = 0.0006). *P*-values are based on a 1-tailed Student’s *t*-test. c) The distribution of viable spores within tetrads is shown for the indicated strains.

To assess the influence of histones H2A.Z and H1 on meiosis in budding yeast, we measured sporulation efficiency, spore viability, and recombination in diploid strains lacking *HHO1*, *HTZ1*, or both (Table 2). We measured the sporulation efficiency by calculating the percentage of diploids that formed spores (either dyads, triads, or tetrads) when cells were placed in media that induce sporulation. Wild-type diploids sporulated at 50% efficiency, similar to prior reports using this strain background (Rockmill and Roeder 1990). Previous studies indicated modest decreases in sporulation efficiency for diploids lacking H2A.Z (Gonzalez-Arranz *et al.* 2018) or H1 (Bryant *et al.* 2012); in our experiments, we observed sporulation efficiencies equivalent to wild-type cells in strains missing H2A.Z, H1, or both (Fig. 1a and Table 2).

**Table 2.** Sporulation efficiency and viability; nondisjunction frequency.

Genotype	Sporulation Efficiency % (*n*) [*P*-value]	% spore viability	# total tetrads analyzed	# 4—Spore viable tetrads	# 3—Spore viable tetrads	# 2—Spore viable tetrads	# 1—Spore viable tetrads	# 0—Spore viable tetrads	Chromosome III nondisjunction (NDJ) %	# NDJ/# 2 spore viable
Wild type	50 (6,800)	96	584	503	64	15	2	0	n.d.	0/15
*htz1Δ/htz1Δ*	48 (6,841) [0.50]	73	781	320	251	102	48	60	0.98	1/102
*hho1Δ/hho1Δ*	56 (6,795) [0.35]	94	643	533	64	41	5	0	n.d.	n.d.
*htz1Δ/htz1Δ* *hho1Δ/hho1Δ*	48 (2,601) [0.24]	81	869	415	284	127	35	8	0.79	1/127

Strain genotypes are described in the first column. Sporulation efficiency is the percentage of diploids that formed spores (either dyads, triads, or tetrads) when cells were placed under sporulation inducing conditions. The second column on the table shows percent sporulation efficiency; the total number of cells analyzed is listed in round brackets, the *p*-value in which each strain is compared to wild-type is listed in round brackets. P-values were calculated using a two-tailed Student's t-test. The third column shows overall percent spore viability by strain as %(total number of spores that germinated/total number of spores analyzed). The fourth column lists the total number of tetrads analyzed for the spore viability experiment. Columns five through nine list the distribution of tetrad types based on number of spores that germinated per dissected tetrad. The last two columns list the frequency of chromosome III non-disjunction events by genotype; n.d., not determined.

Spore viability is expressed as the number of spores that germinate and grow into a colony divided by the total number of spores examined. Wild-type diploids show high spore viability (96%; Fig. 1b and Table 2), similar to previously published values (Sym *et al.* 1993). The spore viability of *hho1Δ*/*hho1Δ* diploids is similar to that of the wild-type strain (94%), consistent with prior reports (Bryant *et al.* 2012; Brush 2015). We observed that deleting *HTZ1* significantly reduced spore viability (73%), in agreement with a prior study (Gonzalez-Arranz *et al.* 2018). Interestingly, deleting *HHO1* in the *htz1Δ* strain caused a statistically significant improvement in spore viability (from 73% to 81%; *P* = 0.0006).

In strains lacking H2A.Z, only 40% of the tetrads contained 4 viable spores, vs >83% in wild-type cells or in cells lacking *HHO1* (Fig. 1c and Table 2). H2A.Z promotes chromosome stability during vegetative growth (Krogan *et al.* 2004; Keogh *et al.* 2006; Sharma *et al.* 2013), and reduced spore viability is often a sign of defective chromosome segregation during either the meiosis I or meiosis II division. To investigate whether the reduced spore viability defect in the *htz1Δ* strain is due to chromosome missegregation during the first meiotic division, we measured the rate of chromosome III nondisjunction events in MI. Nondisjunction of any single chromosome during meiosis I could lead to tetrads with 2 viable spores; if chromosome III undergoes nondisjunction at meiosis I and a normal meiosis II chromosome segregation pattern follows, then each of the 2 viable spore products will express both *MATa* and *MATα* and thus exhibit a nonmating phenotype. The percentage of 2-spore viable tetrads was elevated in the *htz1Δ* mutants relative to wild type (13% vs 2.6%), but the incidence of nonmating 2-spore viable tetrads was not different between the 2 strains (Table 2). Thus, we fail to find the evidence of chromosome III nondisjunction during the first meiotic division. The large increase in 3-spore viable tetrads we observed in the *htz1Δ* mutants (32% in *htz1Δ* vs 11% in wild type) suggests instead that chromatid nondisjunction events during the second meiotic division as a result of precocious sister chromatid separation may be the basis for the decreased spore viability of *htz1Δ* mutants (Rockmill *et al.* 2006; Chu and Burgess 2016).

### Histones H2A.Z and H1 are not required for efficient meiotic recombination

**Fig. 2.**
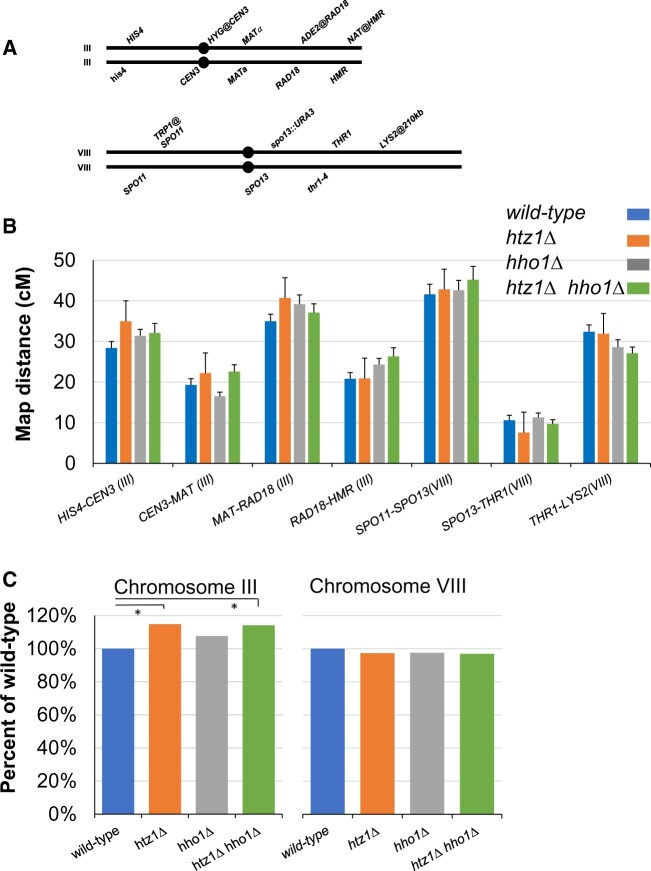
Meiotic recombination frequency in *hho1Δ/hho1Δ* and *htz1Δ/htz1Δ* strains. a) Genetic markers used to assess recombination efficiency on chromosomes III and VIII. b) Recombination frequency. Map distances between the markers shown in (a) were determined. Only 4-spore viable tetrads with no more than 2 gene conversions were used for this analysis. The number of tetrads analyzed: wild type (*n* = 492); *htz1Δ* (*n* = 312); *hho1Δ* (*n* = 525); and *htz1Δ hho1Δ* (*n* = 372). Statistical analysis was performed using the Stahl Lab Online Tools (https://elizabethhousworth.com/StahlLabOnlineTools/) to generate recombination efficiencies, crossover interference and standard error (Stahl and Lande 1995). Error bars represent 1 standard error. Individual recombination values for each interval are listed in Table 3. No statistically significant interval-specific effects are observed in strains lacking either H2A.Z or H1. c) Cumulative recombination frequency on chromosomes III and VIII, relative to wild type. Error bars represent one standard error. Recombination is significantly elevated on chromosome III in the *htz1Δ* strain (*P =* 0.040) and *htz1Δ hho1Δ* strain (*P =* 0.0049). A paired 1-tailed *T*-test was used to assess significance of differences by comparing the *htz1Δ* strains to the wild type.

Meiotic recombination is initiated by the induction of programmed double-strand breaks that are repaired via homologous recombination. To determine if H2A.Z and H1 influence meiotic recombination, we measured recombination frequency across 7 intervals located on chromosomes III and VIII (Fig. 2a) using linkage analysis in strains carrying heterozygous alleles at 9 genetic loci. Our results for the wild-type strain closely match published values *(*Fig. 2b;Voelkel-Meiman *et al.* 2016). Consistent with a prior study assessing 4 intervals on chromosome XV (Brush 2015), we observe no significant changes in recombination rates on chromosomes III or VIII in cells lacking histone H1. Similarly, we observed no significant change in recombination on chromosome VIII due to the loss of H2A.Z, consistent with a prior report examining a single interval on chromosome VIII (Gonzalez-Arranz *et al.* 2018). However, we observed mildly elevated recombination over individual genetic intervals on chromosome III in the absence of H2A.Z (Fig. 2b and Table 3), and cumulatively, we observed that recombination on chromosome III was significantly elevated in both the *htz1Δ* and *htz1Δ hho1Δ* strains (Fig. 2c and Table 3). In wild-type cells, the presence of a crossover decreases the incidence of nearby crossovers (reviewed in Otto and Payseur 2019; von Diezmann and Rog 2021). We found that the individual or combined absence of H2A.Z and H1 did not significantly increase or decrease crossover interference (Table 3).

**Table 3.** Genetic map distances.

Genotype	Interval (chromosome)	PD	TT	NPD	Total	cM (± SE)	% WT	cM by chrm	% WT by chrm	NPDobs/NPDexp (± SE)
Wild type	*HIS4-CEN3 (III)*	230	251	4	485	28.4 (1.6)	100	103.5 (III)	100	0.14 (0.07)
	*CEN3-MAT (III)*	320	165	4	489	19.3 (1.6)	100			0.43 (0.22)
	*MAT-RAD18 (III)*	176	305	6	487	35.0 (1.7)	100			0.11 (0.05)
	*AD18-HMR (III)*	306	179	4	489	20.8 (1.6)	100			0.36 (0.18)
	*SPO11-SPO13(VIII)*	166	301	17	484	41.6 (2.5)	100	84.6 (VIII)	100	0.33 (0.09)
	*SPO13-THR1(VIII)*	386	89	2	477	10.6 (1.2)	100			0.84 (0.60)
	*THR1-LYS2(VIII)*	192	278	5	475	32.4 (1.7)	100			0.12 (0.06)
*htz1Δ/htz1Δ*	*HIS4-CEN3 (III)*	127	168	7	302	34.8 (2.7)	123	118.6(III)	115	0.32 (0.13)
	*CEN3-MAT (III)*	181	125	2	308	22.2 (1.9)	115			0.22 (0.16)
	*MAT-RAD18 (III)*	87	214	6	307	40.7 (2.5)	116			0.22 (0.06)
	*AD18-HMR (III)*	180	129	0	309	20.9 (1.4)	101			n.d.
	*SPO11-SPO13(VIII)*	114	176	14	304	42.8 (3.5)	103	82.24(VIII)	97	0.57 (0.17)
	*SPO13-THR1(VIII)*	257	46	0	303	7.6 (1.0)	72			n.d.
	*THR1-LYS2(VIII)*	119	180	2	301	31.9 (1.9)	98			0.07 (0.05)
*hho1Δ/hho1Δ*	*HIS4-CEN3 (III)*	215	290	5	510	31.4 (1.6)	111	111.33(III)	108	0.13 (0.06)
	*CEN3-MAT (III)*	349	172	0	521	16.5 (1.0)	85			n.d.
	*MAT-RAD18 (III)*	186	312	15	513	39.2 (2.3)	112			0.30 (0.09)
	*AD18-HMR (III)*	285	226	4	515	24.3 (1.5)	117			0.21 (0.11)
	*SPO11-SPO13(VIII)*	171	326	19	516	42.6 (2.4)	102	82.59(VIII)	98	0.32 (0.08)
	*SPO13-THR1(VIII)*	391	107	1	499	11.3 (1.1)	107			0.30 (0.30)
	*THR1-LYS2(VIII)*	247	242	7	496	28.6 (1.8)	88			0.29 (0.11)
*htz1Δ/htz1Δ hho1Δ/hho1Δ*	*HIS4-CEN3 (III)*	166	192	7	365	32.1 (2.4)	113	118.02(III)	114	0.32 (0.13)
	*CEN3-MAT (III)*	211	154	2	367	22.6 (1.7)	117			0.17 (0.12)
	*MAT-RAD18 (III)*	124	234	6	364	37.1 (2.1)	106			0.13 (0.06)
	*AD18-HMR (III)*	195	158	5	358	26.3 (2.2)	126			0.38 (0.18)
	*SPO11-SPO13(VIII)*	125	220	18	363	45.2 (3.3)	109	82.02(VIII)	97	0.51 (0.14)
	*SPO13-THR1(VIII)*	286	69	0	355	9.7 (1.1)	92			n.d.
	*THR1-LYS2(VIII)*	167	186	1	354	27.1 (1.5)	84			0.05(0.05)

Four spore viable tetrads with no more than two gene conversion events each were used for the measurement of map distances and crossover interference as described in (Voelkel-Meiman *et al*., 2015). The specific intervals investigated for recombination efficiency are listed; the number of parental ditypes (PD), tetratypes (TT) and nonparental ditypes (NPD), map distance (in centimorgans; cM), the associated percentage of wild-type for individual intervals and for the entire chromosomes (chrm). Interference is represented on the table as the ratio of observed (obs) to the expected (exp) NPD tetrads. (n.d.) means crossover interference could not be determined due to lack of nonparental ditype (NPD) tetrads.

**Fig. 3.**
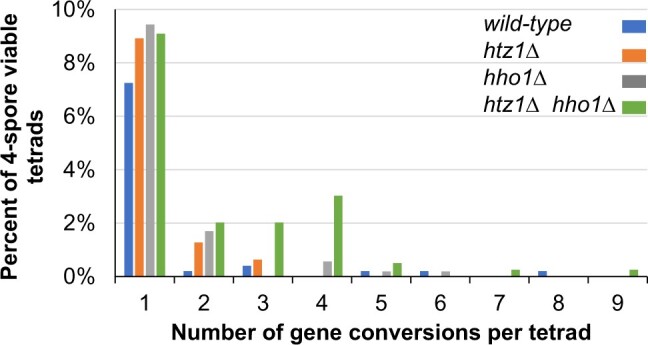
Histones H2A.Z and H1 influence meiotic gene conversion. Gene conversion was tabulated by scoring the number of genetic markers showing non-Mendelian segregation pattern for each tetrad that gave rise to 4 viable spores. The number of tetrads exhibiting the indicated number of gene conversions for each strain is shown on the *x*-axis while frequency of those gene conversions is indicated on the *y*-axis. The total number of tetrads analyzed, number of tetrads containing at least one gene conversion event, and *P*-value by strain are: wild type (*n* = 497, 42); *htz1Δ* (*n* = 314, 34, *P* = 0.26); *hho1Δ* (*n* = 530, 64, *P* = 0.056); and *htz1Δ hho1Δ* (*n* = 396, 68, *P* = 0.001). The raw data used to calculate frequency of gene conversions by genotype are in Table 4.

Meiotic gene conversion results in the non-Mendelian segregation of genetic markers, detectable by observing non-2:2 segregation patterns for heterozygous markers in 4-spore viable tetrads. We observed that the frequency of tetrads experiencing at least 1 gene conversion event is slightly elevated in strains lacking *HHO1* or *HTZ1*, and more markedly in strains lacking both genes (Fig. 3 and Table 4). In addition, we observed an increase in the overall rate of gene conversions per tetrad of 1.3-times in the *hho1Δ* strain and 2.9-times in the *hho1Δ htz1Δ* double mutant. Interestingly, this increase is almost exclusively due to increased gene conversion of chromosome III markers (Table 4; Supplementary Table 2 shows the frequency of gene conversion at individual loci). If we consider the *hho1Δ* and *htz1Δ hho1Δ* tetrads with exactly 2 gene conversions (17 events), we find that the gene converted markers are not more likely to be adjacent to one another than is expected by chance, suggesting that the elevated rates of gene conversion in these strains are due to an increase in the number of interhomolog (noncrossover) recombination events, vs extended gene conversion tracts spanning multiple markers.

**Table 4.** Frequency of gene conversion in strains lacking H2A.Z and H1.

Genotype	# 4—Spore viable tetrads	# tetrads with ≥1 gene conversion (GC)	% tetrads with ≥1 GC	*P*-Value	Total GCs	GCs per tetrad	*P*-Value	GCs per tetrad versus wild type	% total GCs chrom III	% total GCs chrom VIII	# tetrads without a GC	*P*-Value
Wild type	497	42	8	N.A.	63	0.13	N.A.	1	44	56	455	N.A.
*htz1Δ/htz1Δ*	314	34	11	0.26	42	0.13	0.77	1	36	64	280	0.2675
*hho1Δ/hho1Δ*	530	64	12	0.056	91	0.17	0.044	1.3	45	55	466	0.0645
*htz1Δ/htz1Δ hho1Δ/hho1Δ*	396	68	17	0.0001	150	0.38	<0.0001	2.9	75	25	328	<0.0001

Only four spore viable tetrads were used for the analysis of gene conversions (GC). Percentage of tetrads carrying at least one GC was slightly elevated in the absence of H1 and H2A.Z. We used the two proportions Z-test to analyze the significance of difference in GC frequency by comparing the knockout strains to the wild-type. Total number of GC for each strain was used to calculate GC per tetrad. The *hho1Δ and htz1Δ hho1Δ* strains have significantly more GC per tetrad compared to the wild-type. The last column shows Fisher's exact test comparing gene conversion versus non-gene conversion tetrads per strain. Each strain was compared to the wild-type; N.A., not applicable.

### Some *htz1Δ* meiocytes fail to establish a discrete chromosome axis

**Fig. 4.**
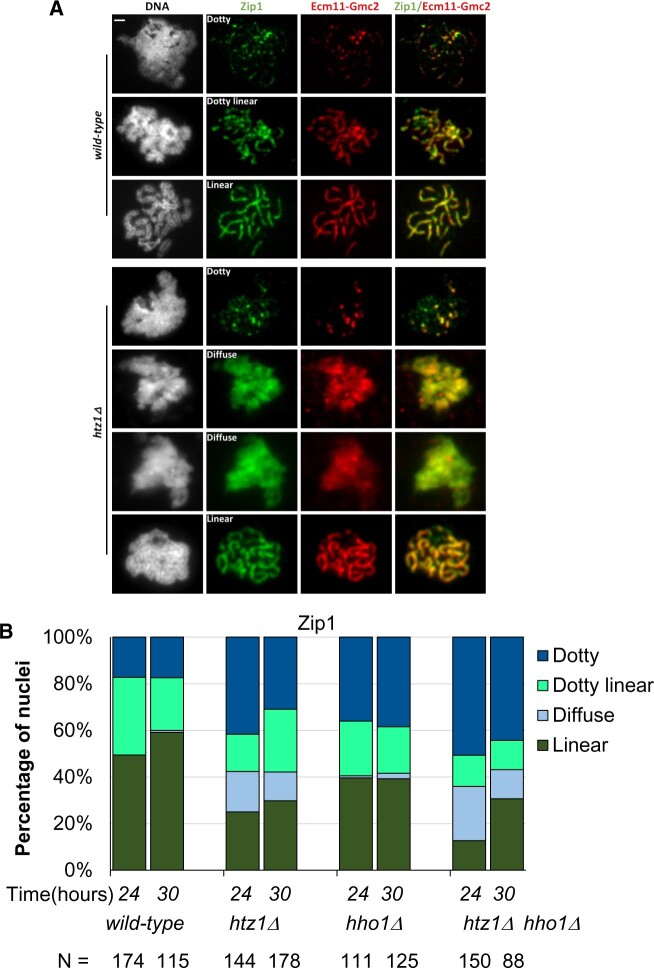
A subset of cells lacking H2A.Z have SC defects. a) Meiotic cell nuclei were surface-spread on glass slides, fixed and stained for the Zip1 and Ecm11-Gmc2 SC proteins (the antibody targeting Ecm11-Gmc2 was raised against a complex of the 2 proteins). Strains examined are homozygous for *ndt80Δ* (Xu *et al.* 1995) and thus fail to progress beyond a late prophase stage of meiosis when SC structures are normally full length. Representative images for wild-type and *htz1Δ* cells are shown. Images for the *hho1Δ* strain are shown in Supplementary Fig. 1. Nuclei were categorized based on Zip1 or Ecm11-Gmc2 staining: linear, long continuous linear structures; dotty linear, a mixture of foci and short linear structures; dotty nuclei, only foci of Zip1, diffuse, a disorganized distribution that is coincident with DAPI. All strains used for cytological analysis are homozygous *ndt80Δ::LEU2*, causing arrest at pachytene of prophase I. Scale bar is 1 µm. b) Distribution of SC structures in all experimental diploids based on Zip1 staining. At least 100 nuclei were analyzed for each strain, from at least 3 independent experiments. Time (*x*-axis) is time post meiotic induction. The number of nuclei assessed for each strain is indicated. An analysis of Ecm11-Gmc2 staining is shown in Supplementary Fig. 2.

The SC is a conserved structure that mediates the alignment of homologous chromosomes during meiotic prophase, serving as the physical context for a large subset of meiotic recombination events (von Wettstein 1984; Zickler and Kleckner 1999; Page and Hawley 2003). The SC has a role in stabilizing paired homologs and regulating crossover formation (Page and Hawley 2003; Voelkel-Meiman *et al.* 2015, 2019). To examine the influence of H2A.Z and H1 on SC assembly, we performed cytological analysis of meiotic chromosomes on cells arrested at the pachytene stage of prophase I. Chromosomes were surface spread at 24 and 30 hr after their introduction into sporulation media and then immunolabeled using antibodies against the SC transverse filament protein, Zip1, and antibodies that target the Gmc2 and/or Ecm11 proteins (Fig. 4a). Zip1 forms transverse filaments of the yeast SC (Sym *et al.* 1993; Sym and Roeder 1995; Symington *et al.* 2014) while Ecm11 and Gmc2 localize to the central element of the budding yeast SC (Humphryes *et al.* 2013; Voelkel-Meiman *et al.* 2013). We assigned nuclei stained for Zip1 protein to one of 4 categories: linear, diffuse, dotty linear, and dotty (illustrated in Fig. 4a and tabulated in Fig. 4b). All strains are homozygous *ndt80Δ::LEU2*, allowing for the enrichment of cells at the pachytene stage of meiosis. At 24 hr postmeiotic induction, the majority of meiotic nuclei of the wild-type strain exhibit extensive linear Zip1 linear structures that are coincident with linear Ecm11-Gmc2 and colocalized with the axes of DAPI-labeled chromosomes, as previously reported (Voelkel-Meiman *et al.* 2013). A minority of wild-type cells show a dotty linear or dotty Zip1 distribution on pachytene chromosomes, where Zip1 structures also coincide with the central element proteins Ecm11-Gmc2. The Zip1 and Ecm11-Gmc2 staining pattern and distribution of the linear and nonlinear classes are not significantly altered in the strain lacking histone H1 (Fig. 4b and Supplementary Fig 1). However, while full-length SC structures were observed in some *htz1Δ* meiotic nuclei, the fraction with mature SC structures is significantly reduced in this strain (Fig. 4b), indicating a defect or delay in SC assembly. A fraction of cells lacking H2A.Z exhibits an unusual SC morphology that we termed “diffuse” (Fig. 4a). Nuclei that display the diffuse phenotype exhibit Zip1 and Ecm11-Gmc2 staining that is uniformly coincident with the DAPI-labeled DNA, but in a disorganized, diffuse pattern. Nuclei with a diffuse phenotype are rare or absent in the wild-type and *hho1*Δ strains (Fig. 4b). A similarly diffuse distribution of Ecm11-Gmc2 is found in this category of nuclei (Supplementary Fig. 2). In contrast to the SC proteins we examined, the non-SC protein tubulin exhibited discrete, specific staining in these chromosome spreads (Supplementary Fig. 3). Cells lacking histone H1 exhibit Zip1 and Ecm11-Gmc2 staining patterns indistinguishable from wild-type cells (Fig. 4b and Supplementary Fig. 1), and we observe that loss of H1 does not suppress or exacerbate the diffuse phenotype caused by the loss of H2A.Z (Fig. 4b).

**Fig. 5.**
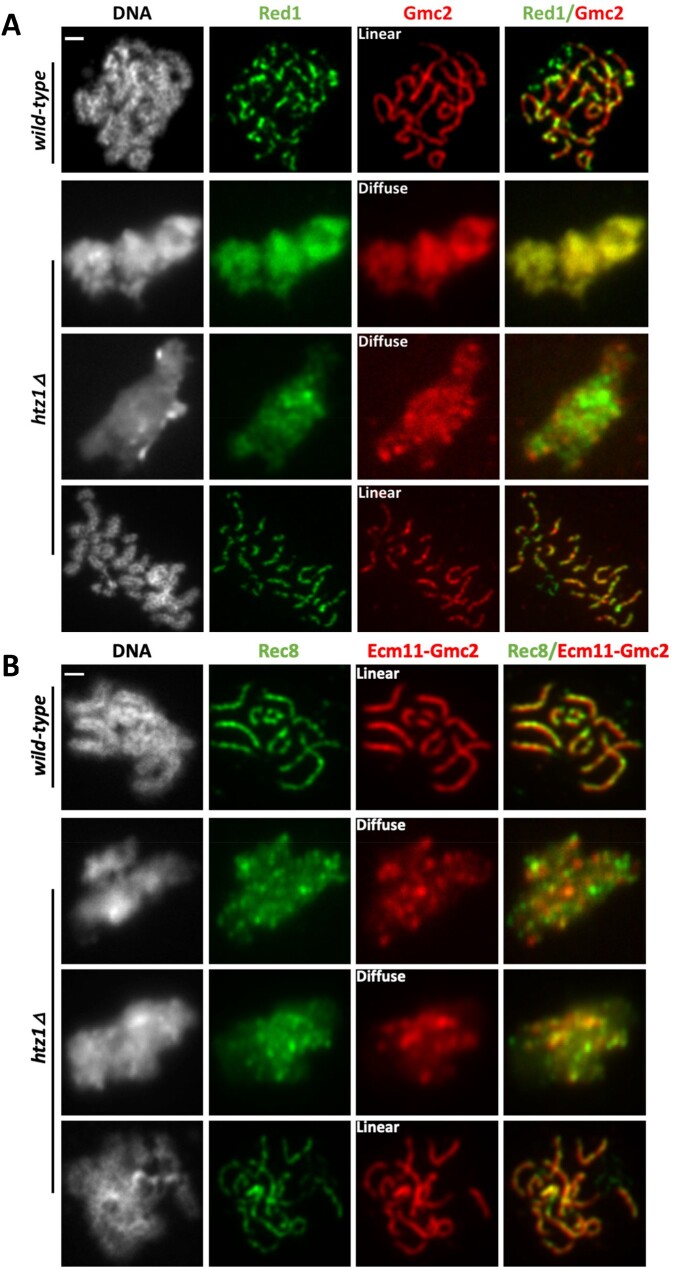
H2A.Z promotes meiotic chromosome axis assembly or maintenance. a) A subset of cells lacking H2A.Z has defective chromosome axes. Meiotic chromosomes were surface-spread at 24 hr after introduction into sporulation media, and labeled with antibodies targeting Red1 and Gmc2. Representative images show the different categories used to assess the state of the SC based on Gmc2 staining, as defined as in the legend of Fig. 4a. b) Representative images for chromosomes stained for Rec8-Myc and Ecm11-Gmc2. Scale bar is 1 µm.

Formation of the SC depends on the prior establishment of a chromosome axis scaffold, formed in part by the Rec8, Red1, and Hop1 proteins (Hollingsworth *et al.* 1990; Smith and Roeder 1997; Klein *et al.* 1999). To investigate the influence of H2A.Z on meiotic chromosome axis formation, we analyzed Red1 and Rec8 localization in cells progressing through meiosis (Fig. 5, a and b and Supplementary Fig. 4). We observe that Red1 partially coincides with Zip1 and Gmc2 in wild-type cells, but is not as continuous, as previously observed (Klein *et al.* 1999; West *et al.* 2019; see top row of Fig. 5a). Cells lacking histone H1 exhibit Red1 and Rec8 staining patterns indistinguishable from wild-type cells (Supplementary Figs. 5 and 6). In the majority of cells lacking H2A.Z, Red1 staining appears indistinguishable from wild-type cells (Fig. 5a). However, in cells exhibiting a diffuse Gmc2 distribution pattern, Red1 exhibits the same diffuse distribution pattern. Rec8 is a meiosis-specific subunit of the cohesion complex important for axis formation (reviewed in Govin and Berger 2009; Neiman 2011; Hunter 2015; Jaiswal *et al.* 2017; Hong *et al.* 2019). We used a strain heterozygous for a *REC8-MYC* allele to observe Rec8 localization. In wild-type meiotic prophase cells, Rec8-Myc is predominantly dotty linear (Fig. 5b), as previously observed (Klein *et al.* 1999; Challa *et al.* 2019). In the absence of H2A.Z, a fraction of cells exhibit a diffuse Rec8-Myc pattern, in which the staining is uniformly coincident with DAPI-labeled DNA and fails to form distinct foci (Fig. 5b). In these nuclei, Rec8 is coincident with the diffusely distributed Ecm11-Gmc2 (Fig. 5b). The disorganized Rec8 observed in a subset of *htz1Δ* meiocytes suggests that failed cohesion function early in meiosis contributes to disorganized chromosomes that fail to properly form chromosome axes and SC.

## Discussion

Chromatin structure influences various aspects of the meiotic program. Here, we have extended earlier studies that established unique roles of the linker histone H1 and variant histone H2A.Z in progression through meiosis. Prior work identified a role for histone H1 in inhibiting homologous recombination in mitotic cells, both in the context of double-strand break repair (Downs *et al.* 2003) and in the suppression of recombination at the rDNA repeats (Li *et al.* 2008). However, initial experiments examining meiotic recombination over 4 intervals on chromosome XV failed to observe changes in meiotic recombination frequency in diploids lacking histone H1 (Brush 2015). We measured recombination over 7 intervals on chromosomes III and VIII in cells lacking H1 and did not observe significantly altered recombination frequencies, suggesting that H1 does not regulate homologous recombination in meiotic cells (Fig. 2b). Consistent with prior experiments examining a single interval on chromosome VIII (Gonzalez-Arranz *et al.* 2018), we also observe no significantly altered recombination frequencies over 7 distinct intervals in strains lacking histone H2A.Z. However, *htz1Δ* cells exhibit an elevated cumulative increase in recombination across chromosome III. We also observed a chromosome III-specific increase in gene conversion in strains lacking H2A.Z. These data suggest that H2A.Z impacts chromosome III structure in a manner that regulates either meiotic recombination initiation (DNA double-strand break formation) or the likelihood of a meiotic double-strand break engaging the homologous chromosome for repair. Interestingly, Lee *et al.* (2021) have recently reported chromosome III-specific decreases in recombination in yeast strains lacking Gmc2 or Ecm11. Distinct features of chromosome III include its relatively small size, its participation in cell type-specific recombination to mediate mating type switching, and the presence of the *HML* and *HMR* heterochromatic regions. One or more of these properties may influence recombination.

In agreement with a prior report (Gonzalez-Arranz *et al.* 2018), we observed a significant decrease in spore viability in strains lacking H2A.Z. Decreased spore viability is frequently a consequence of defects in chromosome segregation. Our failure to observe an increase in nonmating spores in 2-spore viable tetrads suggests that the decrease in viability is not due to nondisjunction in meiosis I. Instead, the high number of 3-spore viable tetrads (Fig. 1c and Table 2) is consistent with premature sister chromatid separation in MI or chromosome nondisjunction during MII (Rockmill *et al.* 2006; Chu and Burgess 2016). H2A.Z is required to promote chromosome stability in budding yeast during vegetative growth (Krogan *et al.* 2004; Keogh *et al.* 2006; Sharma *et al.* 2013). Vegetatively growing cells lacking histone H2A.Z have defects in chromosome cohesion (Sharma *et al.* 2013) and condensation (Rogers and Holmes, unpublished). Compromised condensin protein function causes meiotic defects, including decreased axial compaction (Yu and Koshland 2003; Brito *et al.* 2010; Li *et al.* 2014). It was recently shown that H2A.Z localizes to the spindle pole body and promotes normal chromosome movement during budding yeast meiosis (Gonzalez-Arranz *et al.* 2020). Thus, H2A.Z could contribute to chromosome stability in meiosis by multiple mechanisms. Given the role H2A.Z has in maintaining chromosome stability in mitotic cells, some fraction of *htz1Δ* cells are likely aneuploid when entering meiosis (although these cells would not be predicted to cause an increase in 3 spore viable tetrads).

While strains missing only histone H1 have spore viability comparable to wild-type cells (Bryant *et al.* 2012; Brush 2015; this study), we find that eliminating *HHO1* can partially suppress the spore viability defect in *htz1Δ* cells (Fig. 1b). We have observed that the condensation defect observed in mitotic cells lacking H2A.Z can be partially suppressed by also eliminating histone H1 (Rogers and Holmes, unpublished). In addition, spores from budding yeast that lack histone H1 are reported to have slightly larger nuclear area than spores from wild-type cells, suggesting that their chromatin is relatively decondensed (Bryant *et al.* 2012). *Schizosaccharomyces pombe* strains progressing through meiosis in the absence of histone H2A.Z have more compact nuclei than wild-type cells, suggesting an aberrant increase in chromatin condensation (Yamada *et al.* 2018). Thus, histones H1 and H2A.Z may have opposing roles in establishing a condensed genome, and eliminating H1 may alleviate an over-condensation defect caused by H2A.Z loss.

A fraction of diploids lacking H2A.Z fail to establish a discrete meiotic chromosome axis; in these “diffuse” nuclei, chromosomes fail to appear individualized, and chromatin is uniformly decorated by Rec8, Red1, Zip1, and Gmc2 (Figs. 4 and 5). As initially observed in *red1*, *hop1*, *smc3*, and *rec8* mutants, SC assembly requires formation of chromosomal axes (Hollingsworth *et al.* 1990; Rockmill and Roeder 1990; Klein *et al.* 1999; Panizza *et al.* 2011; Sun *et al.* 2015). Rec8 is a component of a meiosis-specific cohesin complex and is required for formation of axial elements, organized loops of chromatin bound by the Rec8, Hop1, and Red1 proteins. The chromosome axis later forms the lateral elements of the mature SC. Mitotic yeast cells lacking histone H2A.Z exhibit chromosome instability (Krogan *et al.* 2004; Keogh *et al.* 2006) and defects in chromosome cohesion (Sharma *et al.* 2013). Interestingly, the diffuse Rec8 and Zip1 staining we observed in some meiotic nuclei lacking H2A.Z resembles the pattern observed in some nuclei when the Esp1 separase is artificially expressed to cause the premature loss of cohesin during yeast meiosis (Yoon *et al.* 2016); thus, the defects in axis formation we observe in a subset of *htz1Δ* cells may stem from defects in the establishment of cohesion in premeiotic S phase. We note that the spore viability defect caused by the absence of H2A.Z is partially rescued by the deletion of *HHO1*; however, H1 loss fails to ameliorate the defects in axis formation causes by H2A.Z loss. H1 loss may suppress an event in MII downstream of the SC formation defect observed in the MI pachytene-blocked cells. Given H2A.Z’s links to chromosome cohesion and condensation, our models for the phenotypes we observed have focused on altered chromosome stability, but other explanations are possible, including alterations in meiotic transcription programs. Further investigation of H2A.Z and H1’s interactions promises to illuminate additional roles of chromatin in mediating meiosis.

H2A.Z is placed into nucleosomes by the SWR1 complex. Prior studies have found H2A.Z-dependent and H2A.Z-independent roles in meiosis (Gonzalez-Arranz *et al.* 2020). It will be interesting to study whether the *swr1Δ* mutant phenocopies the *htz1Δ* diffuse phenotype addition to whether this defect could be rescued by the overexpression of Rec8 meiotic cohesion.

## Data availability

All yeast strains described in this study are available from the authors upon request. The authors affirm that all data necessary for confirming the conclusions of the article are present within the article, figures, and tables.

Supplemental material is available at *G3* online.

## Supplementary Material

jkac128_Figure_S1

jkac128_Figure_S2

jkac128_Figure_S3

jkac128_Figure_S4

jkac128_Figure_S5

jkac128_Supplemental_Figure_Legends

jkac128_Table_S1

jkac128_Table_S2
