# Supplementary figures and images for: Histone variant H2A.Z promotes meiotic chromosome axis organization in Saccharomyces cerevisiae
Source: G3 (Bethesda). 2022 May 24;12(8):jkac128. doi: 10.1093/g3journal/jkac128 (PMC9339299; doi:10.1093/g3journal/jkac128)

## Slide 1
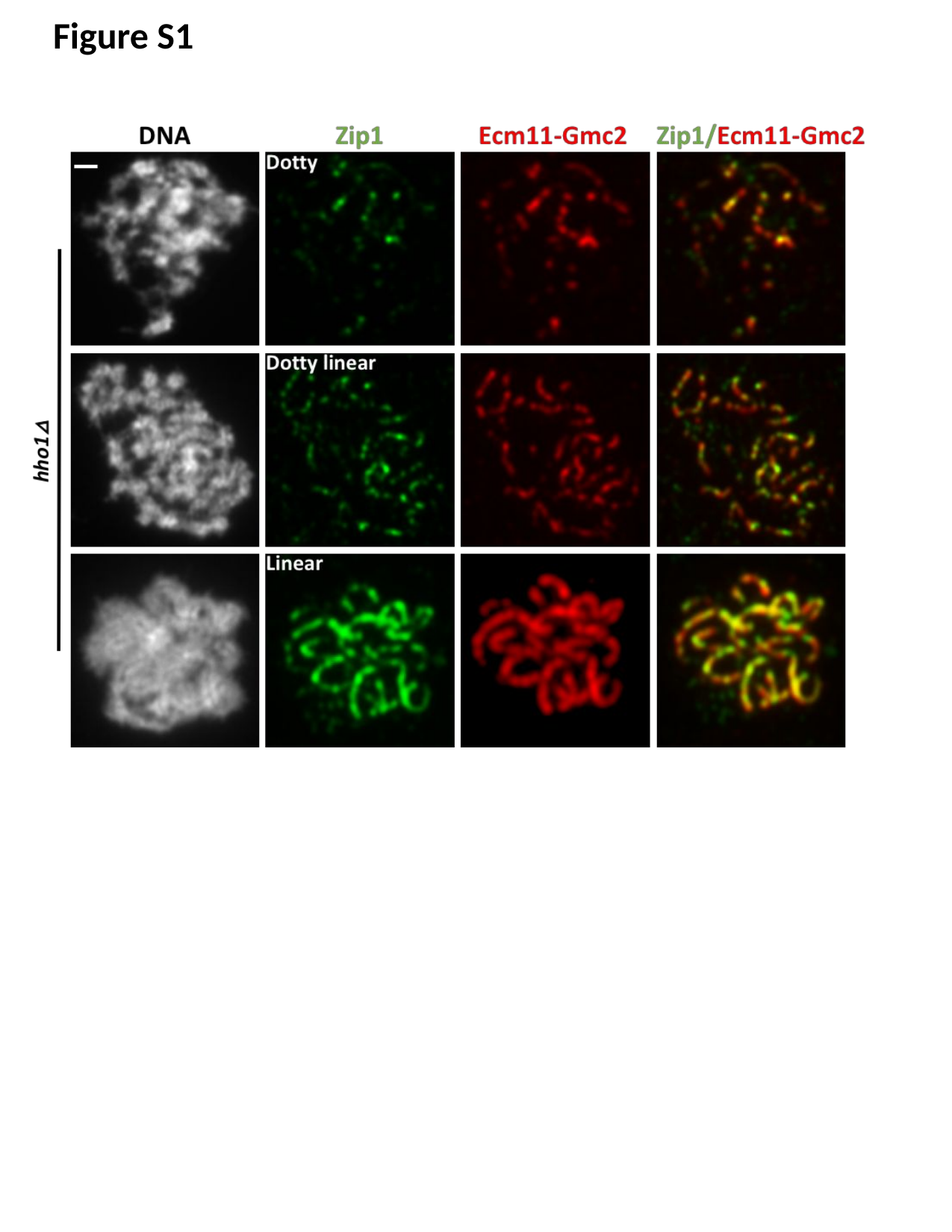

Figure S1

Supplement: jkac128_Figure_S1 [file jkac128_figure_s1.pptx]

## Slide 1
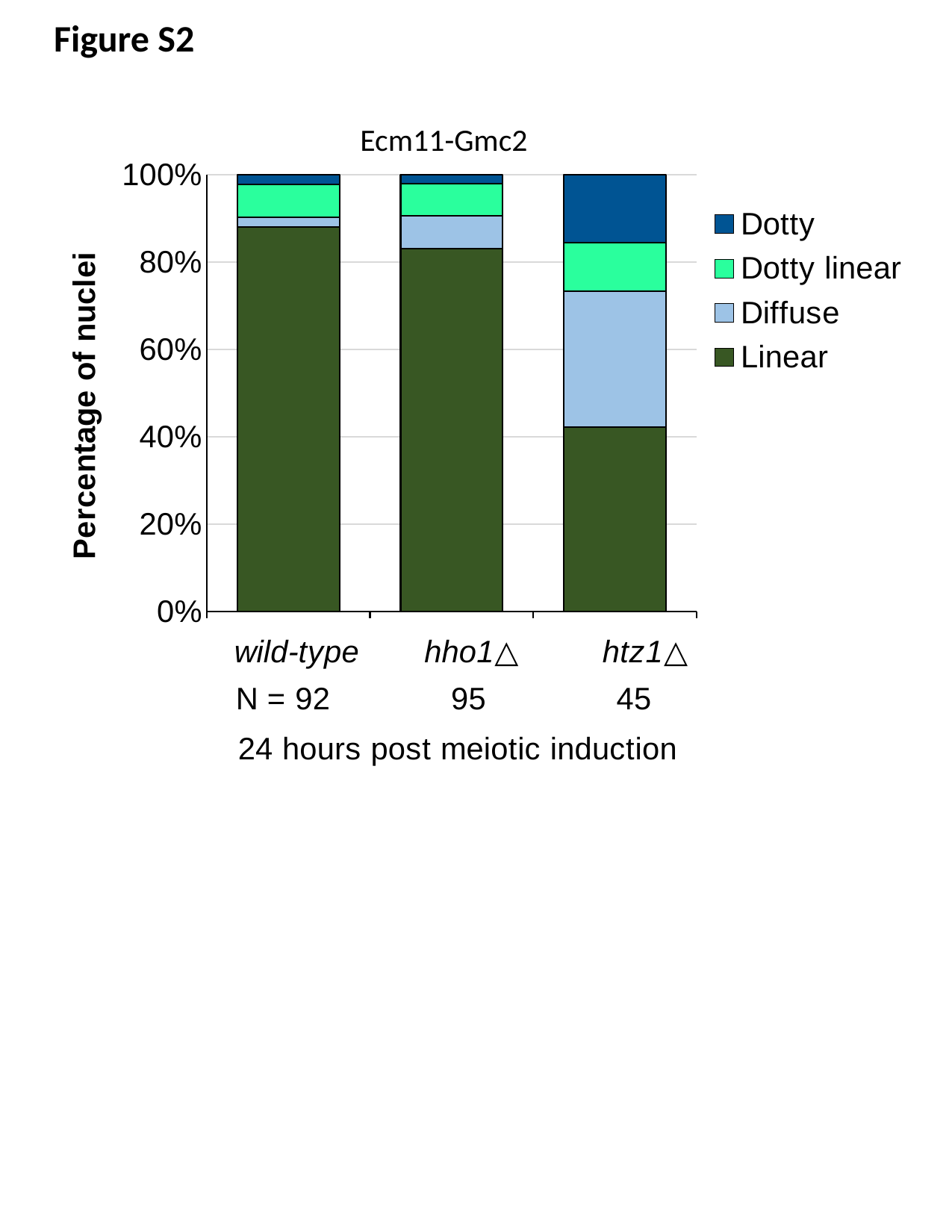

Figure S2
Ecm11-Gmc2
### Chart
| Category | Linear | Diffuse | Dotty linear | Dotty |
|---|---|---|---|---|
| wild-type | 88.04347826086959 | 2.173913043478261 | 7.608695652173914 | 2.173913043478261 |
| hho1∆ | 83.15789473684211 | 7.368421052631578 | 7.368421052631578 | 2.105263157894737 |
| htz1∆ | 42.22222222222222 | 31.11111111111111 | 11.11111111111111 | 15.55555555555556 |

Supplement: jkac128_Figure_S2 [file jkac128_figure_s2.pptx]

## Slide 1
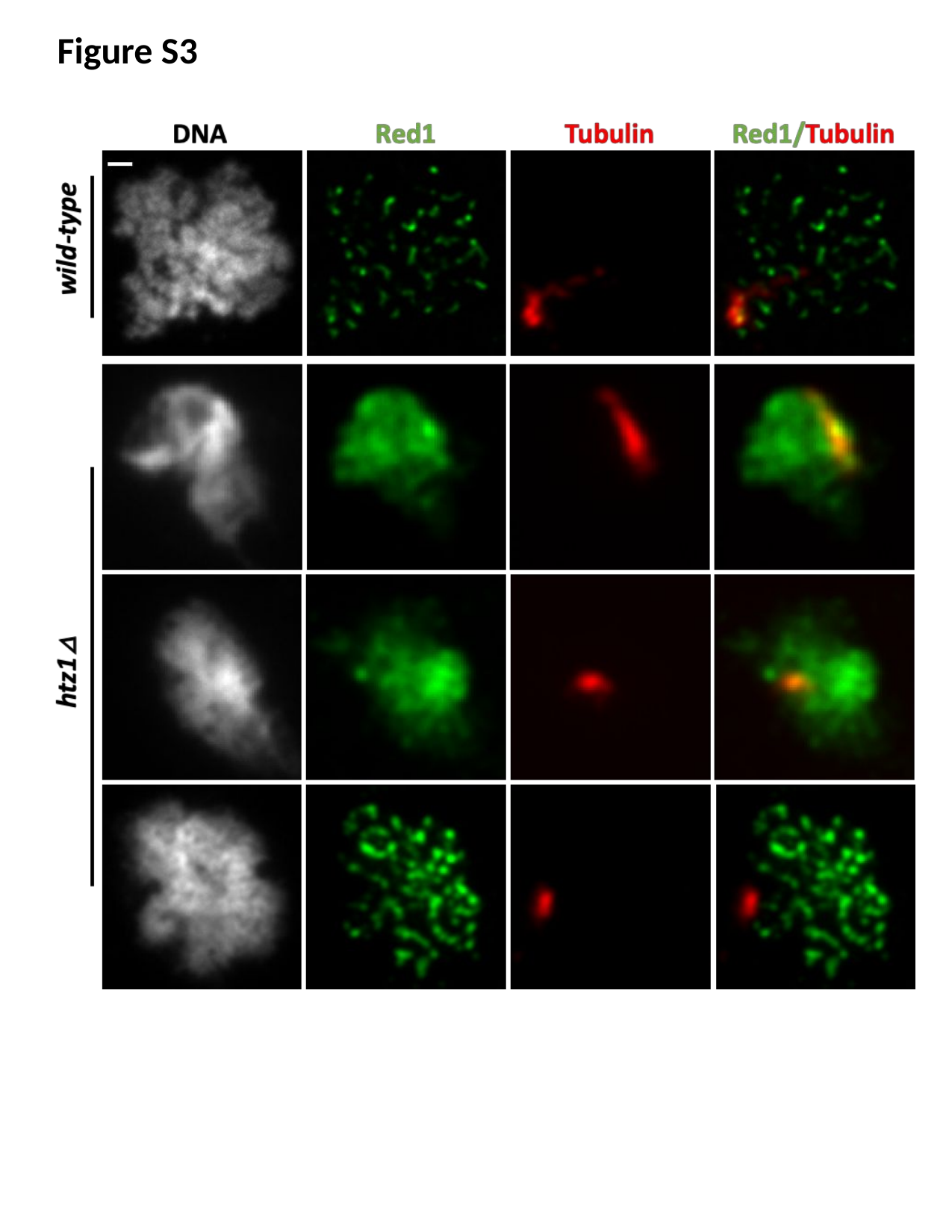

Figure S3

Supplement: jkac128_Figure_S3 [file jkac128_figure_s3.pptx]

## Slide 1
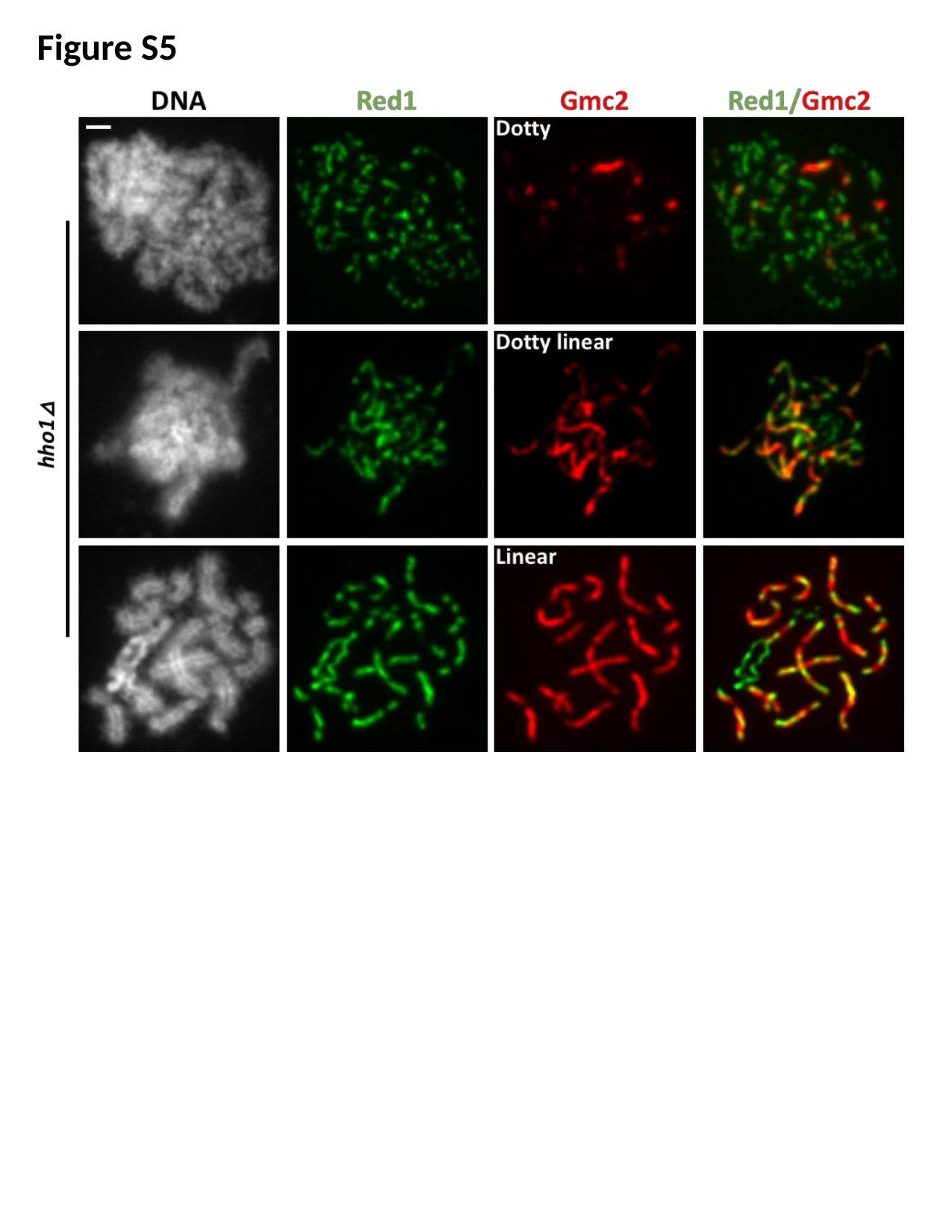

Figure S5

Supplement: jkac128_Figure_S5 [file jkac128_figure_s5.pptx]
