# Supplementary material for: Histone variant H2A.Z promotes meiotic chromosome axis organization in Saccharomyces cerevisiae
Source: G3 (Bethesda). 2022 May 24;12(8):jkac128. doi: 10.1093/g3journal/jkac128 (PMC9339299; doi:10.1093/g3journal/jkac128)
Supplement: jkac128_Figure_S4 [file jkac128_figure_s4.pptx]

## Slide 1
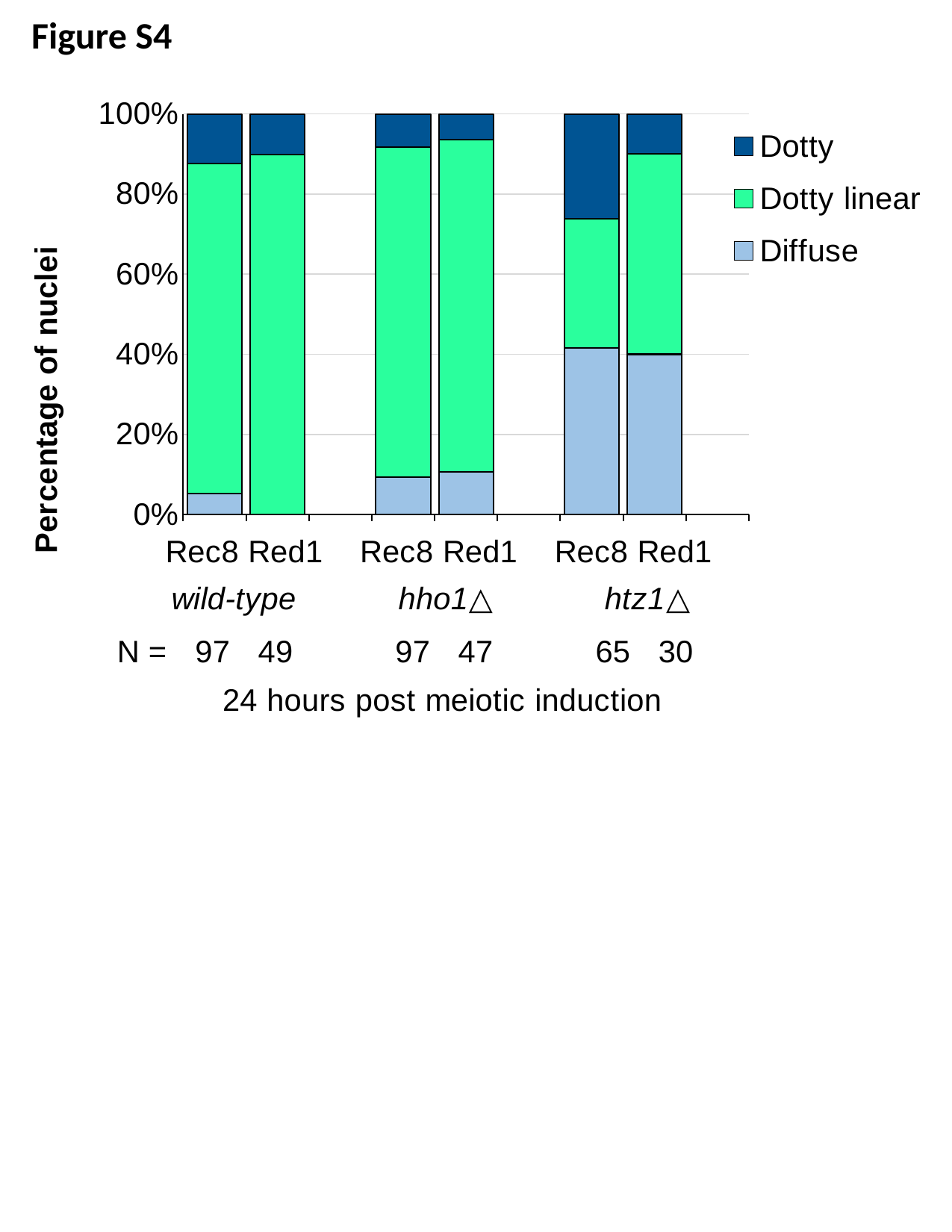

Figure S4
### Chart
| Category | Diffuse | Dotty linear | Dotty |
|---|---|---|---|
| wild type_Rec8 | 5.154639175257731 | 82.4742268041237 | 12.3711340206186 |
| wild type_Red1 | 0.0 | 89.79591836734694 | 10.20408163265306 |
| | None | None | None |
| hho1∆_Rec8 | 9.27835051546392 | 82.4742268041237 | 8.247422680412367 |
| hho1∆_Red1 | 10.63829787234042 | 82.97872340425533 | 6.382978723404255 |
| | None | None | None |
| htz1∆_Rec8 | 41.53846153846154 | 32.30769230769229 | 26.15384615384616 |
| htz1∆_Red1 | 40.0 | 50.0 | 10.0 |
