# Supplementary material for: Histone variant H2A.Z promotes meiotic chromosome axis organization in Saccharomyces cerevisiae
Source: G3 (Bethesda). 2022 May 24;12(8):jkac128. doi: 10.1093/g3journal/jkac128 (PMC9339299; doi:10.1093/g3journal/jkac128)
Supplement: jkac128_Supplemental_Figure_Legends [file jkac128_supplemental_figure_legends.docx]

**Supplementary Figure Legends**

**Figure S1**

Representative images of SC structures in the *hho1Δ* strains based on Zip1 staining. Similar to the wild-type, mostly linear SC structures are observed in the *hho1Δ* strain. Scale bar is 1 μm.

**Figure S2**

Distribution of SC structures based on Ecm11-Gmc2 antibody staining. The same nuclei were also stained for Red1 and Rec8 meiotic proteins. At least 45 nuclei were analyzed for each strain, accumulated from at least three independent experiments. The wild-type and *hho1Δ* strains exhibit mostly linear SC structures. In the *htz1Δ* strain, linear SC structures are reduced by about half compared to wild-type while a new class of “diffuse” nuclei is observed, which display an SC protein distribution coincident with the DAPI-labeled DNA.

**Figure S3**

Representative images of tubulin staining in the wild-type and *htz1Δ* strains. In the wild-type strain, Red1 is dotty linear along the chromosome axes while tubulin forms a single discrete foci. A subset of the *htz1Δ* cells exhibit diffuse Red1 staining; in these same cells, 86% exhibit discrete foci of tubulin staining (n = 66). The frequency of observing discrete tubulin foci in cells with diffuse Red1 nuclei was consistent between trials. Scale bar is 1 μm.

**Figure S4**

Evaluation of meiotic chromosome axis structures based on Rec8 and Red1 staining. At least 40 nuclei from at least two independent experiments were analyzed for each strain. In the wild-type and *hho1Δ* strains, both Rec8 and Red1 appear as mostly dotty linear and dotty (foci) structures. In the absence of H2A.Z, dotty linear Rec8 and Red1 structures are highly reduced. In addition, the *htz1Δ* strain has an elevated occurrence of nuclei with a diffuse Rec8 and Red1 distribution, which is rarely found in the wild-type and *hho1Δ* strains. Analysis of individual nuclei show that Rec8 appears diffuse in 100% of nuclei with diffuse Red1, and vice-versa.

**Figure S5**

Representative images of the Red1 staining pattern in the *hho1Δ* strain. Red1 shows a dotty linear or dotty distribution, as is observed in the wild-type. Central element protein Gmc2 was analyzed to assess the state of the SC. Scale bar is 1 μm.

**Figure S6**

Representative images of the meiosis specific cohesion protein Rec8 in the *hho1Δ* strain. Similar to the wild-type strain, Rec8 shows a dotty linear or dotty distribution pattern on meiotic prophase chromosomes. Central element proteins Ecm11-Gmc2 were analyzed to assess the state of the SC. Scale bar is 1 μm.
