# Supplementary material for: Histone variant H2A.Z promotes meiotic chromosome axis organization in Saccharomyces cerevisiae
Source: G3 (Bethesda). 2022 May 24;12(8):jkac128. doi: 10.1093/g3journal/jkac128 (PMC9339299; doi:10.1093/g3journal/jkac128)
Supplement: jkac128_Table_S1 [file jkac128_table_s1.docx]

**Table S1. Primers**

| **Primer** | **Primer sequence** | **Description** |
| --- | --- | --- |
| SP470 | AATTCAATTTCGCACTATAGCCGCACGTAAAAATAACTTAACATAcgtacgctgcaggtcgac | Forward primer for deleting *HTZ1* gene and replacing it with *MX* marker |
| SP471 | GGAGCAGGGAGAATTACGGGAAATGGGAAAGAAAAACTATTCTTCatcgatgaattcgagctcg | Reverse primer for deleting *HTZ1* gene and replacing it with *MX* marker |
| SP472 | TTACTGAATGCATCCATGC | Check primer for confirming *HTZ1* gene deletion |
| SP101 | GTGATGTGAGAACTGTATCC | Check primer for confirming *HTZ1* and *HHO1* gene deletion |
| SP467 | AATGCTTGGCAGCGAGGGAAGCAATTATAATACAACTAAAGCAACcgtacgctgcaggtcgac | Forward primer for deleting *HHO1* gene and replacing it with *MX* marker |
| SP468 | TAGTATTGCTATCACCATTGACATTCTCGTTTGGATATTCACTTTatcgatgaattcgagctcg | Reverse primer for deleting *HHO1* gene and replacing it with *MX* marker |
| SP469 | ATGAAATGCTATTCTGTGTC | Check primer for confirming *HHO1* gene deletion |
