# Supplementary material for: Histone variant H2A.Z promotes meiotic chromosome axis organization in Saccharomyces cerevisiae
Source: G3 (Bethesda). 2022 May 24;12(8):jkac128. doi: 10.1093/g3journal/jkac128 (PMC9339299; doi:10.1093/g3journal/jkac128)
Supplement: jkac128_Table_S2 [file jkac128_table_s2.docx]

**Table S2. Non-mendelian segregation events**

| **% of 3:1/1:3 events** | | | **CHROMOSOME III** | | | | | **CHROMOSOME VIII** | | | |  |  |
| --- | --- | --- | --- | --- | --- | --- | --- | --- | --- | --- | --- | --- | --- |
|  | strain # | # 4-Spore viable tetrads | *HIS4* | *HYG @ CEN3* | *MAT* | *ADE @ RAD18* | *natMX @ HMR* | *TRP1 @ SPO11* | *spo13::* | *HIS4* | *HYG @ CEN3* | **Sum 3:1/1:3** | **Mutant/**  **wild-type**  **fold increase** |
| *wild-type* | YSH1496 | 497 | 1.2 | 0.2 | 0.6 | 0.6 | 0.2 | 1.8 | 0.2 | 3.0 | 0.4 | 8.2 | 1.0 |
| *htz1∆ /htz1∆* | YSH1497 | 314 | 2.9 | 0.6 | 0.6 | 1.0 | 0.3 | 2.5 | 0.3 | 2.9 | 0.6 | 11.8 | 1.4 |
| *hho1∆ /hho1∆* | YSH1498 | 530 | 2.8 | 0.4 | 0.8 | 1.3 | 0.6 | 1.1 | 0.0 | 5.1 | 0.4 | 12.5 | 1.5 |
| *htz1∆ /htz1∆*  *hho1∆ /hho1∆* | YSH1515 | 396 | 5.1 | 1.3 | 1.3 | 5.8 | 5.6 | 3.3 | 0.5 | 4.3 | 1.0 | 28.0 | 3.4 |
| **% of 4:0/0:4 events** | | |  |  |  |  |  |  |  |  |  |  | **Sum 4:0/0:4** |
| *wild-type* | YSH1496 | 497 | 0.6 | 0.6 | 0.2 | 0.8 | 0.6 | 0.4 | 0.2 | 0.4 | 0.6 | 4.4 | 1.0 |
| *htz1∆ /htz1∆* | YSH1497 | 314 | 0.0 | 0.0 | 0.0 | 0.0 | 0.0 | 0.3 | 0.3 | 0.6 | 0.3 | 1.6 | 0.4 |
| *hho1∆ /hho1∆* | YSH1498 | 530 | 0.6 | 0.2 | 0.0 | 0.9 | 0.6 | 0.8 | 0.4 | 0.6 | 0.8 | 4.7 | 1.1 |
| *htz1∆ /htz1∆*  *hho1∆ /hho1∆* | YSH1515 | 396 | 1.0 | 4.8 | 0.3 | 1.0 | 2.5 | 0.0 | 0.3 | 0.0 | 0.0 | 9.8 | 2.2 |
